# Molecular characterization of multiresistant *Escherichia coli* producing or not extended-spectrum β-lactamases

**DOI:** 10.1186/1471-2180-13-84

**Published:** 2013-04-16

**Authors:** Belén Ruiz del Castillo, Laura Vinué, Elena Jesús Román, Beatriz Guerra, Alessandra Carattoli, Carmen Torres, Luis Martínez-Martínez

**Affiliations:** 1 Service of Microbiology, Hospital Universitario Marqués de Valdecilla-IFIMAV, Av/ de Valdecilla s/n, Santander 39008, Spain; 2 Área de Bioquímica y Biología Molecular, Universidad de La Rioja, Logroño, Spain; 3 Federal Institute for Risk Assessment, Berlin, Germany; 4 Laboratory of Bacteriology and Medical Mycology, Istituto Superiore di Sanita, Rome, Italy; 5 Department of Molecular Biology, University of Cantabria, Santander, Spain

**Keywords:** *E. coli*, Plasmids, ESBL, Multiresistance

## Abstract

**Background:**

The prevalence and type of plasmids, resistance genes and integrons carried by two collections of multiresistant *E. coli* producing or not extended-spectrum β-lactamases have been compared. Rep-PCR was used to determine the clonal relationship of the organisms. Plasmids were classified according to their incompatibility. Class 1 and Class 2 integrons and antibiotic resistance genes were analysed by PCR and sequencing.

**Results:**

Both collections of organisms contained a large diversity of unrelated strains with some clones distributed in both groups of isolates. Large plasmids were identified in the two groups of organisms. Plasmids with replicons *repK* and *repColE* were more frequent among ESBL-producing isolates, while *repFIA*, *repFII* and *repA/C* replicons were more frequent in isolates lacking ESBL. Conjugative plasmids with *repK* and *repA/C* replicons coded for CTX-M-14 and CMY-2 β-lactamases, respectively. No significant differences were observed in the distribution of class 1 and class 2 integrons among multiresistant *E. coli* producing or not ESBL, and *dfrA17-ant(3″)-Ie* was the cassette arrangement most commonly found.

**Conclusions:**

In the concrete temporal and geographical context of this study, multiresistant *E. coli* producing ESBL or other mechanisms of resistance were largely clonally diverse and present some differences in the types of harboured plasmids. Still, some clones were found in both ESBL-producing and –lacking isolates.

## Background

A rapid dissemination of *Escherichia coli* and others enterobacteria producing extended-spectrum beta-lactamases (ESBLs) has been reported in many European countries, including Spain, and is a matter of major concern [1,2]. The *bla*_CTX-M_ genes are becoming the most prevalent ESBLs encountered in *Enterobacteriaceae*[2]. The prevalent *bla*_CTX-M_-type genes in Europe have been identified as *bla*_CTX-M-1_, *bla*_CTX-M-3_, *bla*_CTX-M-9_, *bla*_CTX-M-14_ and *bla*_CTX-M-15_[2]. Infections caused by enterobacteria producing ESBL are associated with increased morbidity, mortality, and health-care associated costs [3].

During recent years, extensive characterization of plasmid families (usually by replicon typing [4,5] or, more recently, by relaxase identification [6]) has provided additional information on epidemiological aspects of transmissible antimicrobial resistance [7]. Many of these studies have focused on *E. coli* producing ESBL [8,9]. From these studies, some plasmid families were demonstrated to be largely prevalent in Enterobacteriaceae, emerging in association with specific ESBL genes. For instance, the *bla*_*CTX-M-15*_ and *bla*_*CTX-M-3*_ genes have often been located on plasmids belonging to the IncF group [10] and IncL/M family [11] respectively, in different countries [12-16]. It would be interesting to know if in a specific geographical area, plasmid-mediated antimicrobial resistance in multiresistant *E. coli* producing ESBL is due to plasmids of the same incompatibility group(s) as those present in other multirresistant isolates not producing ESBL.

The objective of this study was to determine whether the clonal variability and content of plasmids, resistance genes and integrons of clinical isolates of *E. coli* producing ESBL (Ec-ESBL) were similar or not to those of *E. coli* isolates lacking ESBL (Ec-MRnoB) isolated in the same geographical area and period.

## Results

### Phenotype of resistance and clonal relationship

MIC_50_ and MIC_90_ values of the different agents against the two groups of multiresistant *E. coli* are presented in Table 1. All Ec-ESBL were susceptible to cefotetan, imipenem, meropenem, amikacin and tigecycline. According to the EUCAST [17], all Ec-ESBL isolates were resistant to cefotaxime, 96% to cefepime, 96% to aztreonam and 23% to ceftazidime (Table 1). Moreover, 39% of the isolates belonging to the Ec-ESBL collection were co-resistant to quinolones, tetracycline and trimethoprim-sulfamethoxazole.

**Table 1:** **MIC range, MIC**_**50 **_**(mg/L) and MIC**_**90 **_**(mg/L) values of 21 antimicrobial agents for Ec-ESBL and Ec-MRnoB isolates, and percentages of resistant isolates to the indicated agents (EUCAST breakpoints, except where indicated)**

	**Ec-ESBL (N=100)**				**Ec-MRnoB (N=100)**			
**Agent**	**Range**	**MIC**_**50**_	**MIC**_**90**_	**%R**	**Range**	**MIC**_**50**_	**MIC**_**90**_	**%R**
**Amoxicillin**	64-≥256	≥256	≥256	100	2-≥256	≥256	≥256	99
**Amoxicillin-Clavulanic acid**	8-16	4	8	41	8-≥128	8	32	83
**Piperacillin**	32-≥64	≥64	≥64	100	≤16-≥64	≥64	≥64	84
**Piperacillin-Tazobactam**	≤0.5-≥256	4	16	16	≤0.25-≥256	2	4	6
**Cefoxitin**^**a**^	≤2-32	≤2	8	5	≤2-≥32	4	8	8
**Cefotetan**^**a**^	≤1-8	≤1	≤1	0	≤1-≥32	≤1	≤1	2
**Cefotaxime**	32-≥128	≥128	≥128	100	≤0.5-128	≤0.5	≤0.5	5
**Ceftazidime**	≤0.5-≥128	2	16	23	≤0.5-128	≤0.5	≤0.5	8
**Cefepime**	≤1-≥32	≥32	≥32	96	≤1-4	≤1	<1	1
**Aztreonam**	2-≥64	16	≥64	96	≤0.5-32	≤0.5	≤0.5	6
**Imipenem**	≤0.5-1	≤0.5	≤0.5	0	≤0.5-2	≤0.5	≤0.5	0
**Meropenem**	≤0.5	≤0.5	≤0.5	0	≤0.5	≤0.5	≤0.5	0
**Gentamicin**	≤0.5-≥256	1	32	19	0.5-≥256	64	256	96
**Tobramycin**	0.5-256	1	8	17	≤0.25-256	8	32	89
**Amikacin**	≤0.5-8	2	4	0	1-8	2	4	0
**Nalidixic acid**^**a**^	1-≥256	≥256	≥256	89	1-≥256	≥256	≥256	98
**Ciprofloxacin**^**a**^	≤0.5-≥256	16	128	74	≤0.25-≥256	32	128	93
**Tetracycline**^**a**^	0.5-≥256	256	256	80	≤0.25-256	256	256	84
**Doxycycline**^**a**^	≤0.5-128	16	64	76	≤0.25-128	32	64	79
**Tigecycline**^**b**^	≤0.5-1	≤0.5	≤0.5	0	≤0.25-0.5	≤0.25	≤0.25	0
**Trimethoprim-Sulfamethoxazole**	≤0.5-≥32	≥32/608	≥32/608	67	≤0.5-≥32	≥32/608	≥32/608	98

^a^ CLSI 2011 breakpoints. ^b^ FDA breakpoints.

All Ec-MRnoB were susceptible to imipenem, meropenem, amikacin and tigecycline. Eight isolates in this collection were resistant to at least one extended-spectrum cephalosporin (ceftazidime) (Table 1). The most frequent phenotype of resistance observed among the selected Ec-MRnoB isolates included resistance to β-lactams (amoxicillin), aminoglycosides [gentamicin alone or (more often) associated to tobramycin], quinolones (nalidixic acid alone or associated to ciprofloxacin), tetracyclines (tetracycline alone or associated to doxycycline) and trimethoprim-sulfamethoxazole, occurring in 50% of the studied isolates. All other possible combinations of co-resistances among the selected isolates represented no more than 5% of the isolates.

Most Ec-ESBL were of phylogroup B1 (38%), followed by groups A (32%), D (22%) and B2 (8%). In contrast, the most frequent phylogenetic group of Ec-MRnoB was D (46%), followed by groups A (25%), B2 (17%) and B1 (12%).

The 100 Ec-ESBL isolates were grouped in 66 Rep-PCR patterns. In this group, only 2 Rep-PCR patterns included 5 or more isolates: patterns XXXI (n=6, phylogenetic group A) and XXII (n=5; B1). The remaining patterns contained 2 to 4 isolates (16 Rep-PCR patterns) or single isolates (48 Rep-PCR patterns). Lower clonal variability was noted among the Ec*-*MRnoB, which were grouped into 40 Rep-PCR patterns. Three patterns included 5 or more isolates: I-NB (n=18, phylogenetic group D), II-NB (n=14; B2) and XXIII-NB (n=8; D). Fifteen patterns included 2 to 4 isolates, and the remaining 22 patterns corresponded to single isolates.

**Figure 1:**
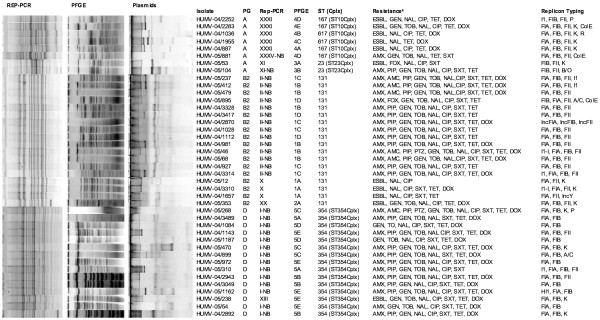
**Clonal relationship and plasmid profile of related isolates from both collections. **^a^ESBL= extended-spectrum β-lactamases; AMX=amoxicillin, PIP= piperacillin; AMC=amoxicillin-clavulanic acid; CAZ=ceftazidime, FOX=cefoxitin; GEN=gentamicin, TOB=tobramycin, NAL=nalidixic acid, CIP=ciprofloxacin, TET= tetracycline, SXT= trimethoprim.-sulphamethoxazole.

Comparison of Rep-PCR patterns corresponding to isolates of the two *E. coli* collections showed the presence of Ec-ESBL (5 Rep-PCR patterns corresponding to 11 isolates) and Ec-MRnoB (4 Rep-PCR patterns corresponding to 30 isolates) with the same pattern. PFGE analysis confirmed this relationship, with isolates presenting identical or similar [less than 5 bands of difference] PFGE patterns. In addition, clonally related isolates (determined by Rep-PCR/PFGE) were included in the same ST defined by MLST (Figure 1).

Twenty-four ST were identified among the isolates selected for the conjugation assay (Table 2), of which seven types included both Ec-ESBL and Ec-MRnoB, while 11 and 6 types included either Ec-ESBL or Ec-MRnoB, respectively. Three new ST (ST2292, ST2294 and ST2295) were first identified among our Ec-ESBL isolates.

**Table 2:** **Clonal complexes (Cplx) and sequence types (ST) of 40 multiresistant *E. coli *producing ESBL (Ec-ESBL, n=20) or not (EcMRnoB, n=20) selected for the conjugation assay**

**Isolate**	***E. coli *collection**	**ST**	**Cplx**	**adk**	**fumC**	**gyrB**	**icd**	**mdh**	**purA**	**recA**	**Rep-PCR**	**Nª**
HUMV-05/374	Ec-MRnoB	ST1011		6	4	159	44	112	1	17	III-NB	3
HUMV-04/2539	Ec-ESBL	ST117		20	45	41	43	5	32	2	V	2
HUMV-05/21	Ec-MRnoB	ST117		20	45	41	43	5	32	2	XXIII-NB	8
HUMV-04/1119	Ec-MRnoB	ST1210		187	11	4	8	8	8	2	IV-NB	3
HUMV-04/925	Ec-MRnoB	ST1210		187	11	4	8	8	8	2	VI-NB	4
HUMV-04/3310	Ec-ESBL	ST131		53	40	47	13	36	28	29	X	3
HUMV-05/46	Ec-MRnoB	ST131		53	40	47	13	36	28	29	II-NB	14
HUMV-05/895	Ec-MRnoB	ST131		53	40	47	13	36	28	29	II-NB	14
HUMV-04/2296	Ec-MRnoB	ST131		53	40	47	13	36	28	29	XII-NB	1
HUMV-04/2830	Ec-ESBL	ST155	ST155Cplx	6	4	14	16	24	8		XXII	5
HUMV-04/2103	Ec-ESBL	ST156	ST156Cplx	6	29	32	16	11	8	44	XXXVII	3
HUMV-04/2283	Ec-ESBL	ST167	ST10Cplx	10	11	4	8	8	13	2	XXXI	6
HUMV-05/868	Ec-MRnoB	ST167	ST10Cplx	10	11	4	8	8	13	2	VI-NB	4
HUMV-05/881	Ec-MRnoB	ST167	ST10Cplx	10	11	4	8	8	13	2	XXXV-NB	1
HUMV-04/2487	Ec-ESBL	ST224		6	4	33	16	11	8	6	XXIV	2
HUMV-04/3181	Ec-MRnoB	ST224		6	4	33	16	11	8	6	X-NB	3
HUMV-04/3218	Ec-MRnoB	ST224		6	4	33	16	11	8	6	XVII-NB	1
HUMV-04/1412	Ec-ESBL	ST2292		6	65	15	18	9	8	6	LIII	1
HUMV-04/1391	Ec-ESBL	ST2296		8	7	1	8	6	8	7	XXII	5
HUMV-05/284	Ec-ESBL	ST2295		8	7	14	8	8	8	6	XVII	3
HUMV-04/3168	Ec-MRnoB	ST23	ST23Cplx	6	4	12	1	20	13	7	XXXIX-NB	2
HUMV-05/268	Ec-MRnoB	ST354	ST354Cplx	85	88	78	29	59	58	62	I-NB	18
HUMV-05/310	Ec-MRnoB	ST354	ST354Cplx	85	88	78	29	59	58	62	I-NB	18
HUMV-05/281	Ec-ESBL	ST359		43	41	15	90	11	8	6	XIV	4
HUMV-05/508	Ec-ESBL	ST359		43	41	15	90	11	8	6	XIV	4
HUMV-04/1087	Ec-ESBL	ST362		62	100	17	31	5	5	4	XXIII	2
HUMV-04/3096	Ec-ESBL	ST393	ST31Cplx	18	106	17	6	5	5	4	III	4
HUMV-05/631	Ec-MRnoB	ST393	ST31Cplx	18	106	17	6	5	5	4	XV-NB	3
HUMV-04/2833	Ec-ESBL	ST538	ST538Cplx	13	40	19	13	36	28	30	IV	3
HUMV-04/1630	Ec-ESBL	ST57	ST350Cplx	6	31	5	28	1	1	2	XXXIX	2
HUMV-05/190	Ec-ESBL	ST617	ST10Cplx	10	11	4	8	8	13	73	II	3
HUMV-04/3317	Ec-ESBL	ST617	ST10Cplx	10	11	4	8	8	13	73	XXXI	6
HUMV-04/2942	Ec-ESBL	ST641	ST86Cplx	9	6	33	131	24	8	7	XVI	2
HUMV-04/1012	Ec-ESBL	ST648		92	4	87	96	70	58	2	LVIII	2
HUMV-05/630	Ec-MRnoB	ST648		92	4	87	96	70	58	2	XXXI-NB	1
HUMV-04/1002	Ec-MRnoB	ST69	ST69Cplx	21	35	27	6	5	5	4	IX-NB	4
HUMV-04/1725	Ec-ESBL	ST88	ST23Cplx	6	4	12	1	20	12	7	XXXVI	2
HUMV-05/569	Ec-MRnoB	ST88	ST23Cplx	6	4	12	1	20	12	7	XXIV-NB	4
HUMV-05/523	Ec-MRnoB	ST88	ST23Cplx	6	4	12	1	20	12	7	XXX-NB	1
HUMV-04/979	Ec-MRnoB	ST93	ST168Cplx	6	11	4	10	7	8	6	XXXVII-NB	1

^a^Total number of isolates belonging to the indicated Rep-PCR pattern.

### Plasmid profile

**Figure 2:**
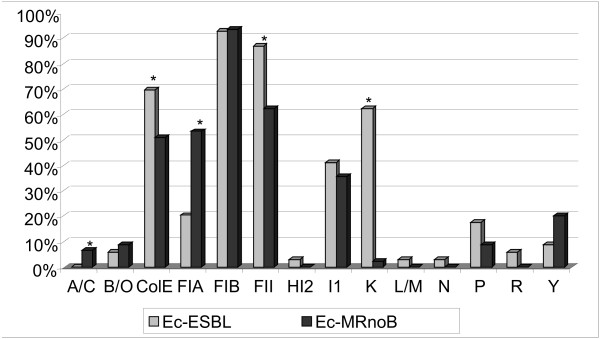
**Distribution of replicons on plasmids identified in multiresistant *E. coli *isolates producing ESBL (Ec-ESBL, n=69) or lacking these enzymes (Ec-MRnoB, n=45).** *p value <0.05.

One or more plasmids were observed in organisms of the two *E. coli* collections. For the 69 Ec-ESBL isolates the most frequently detected replicons were *repFIB* (64/69, 92.8%), r*epFII* (60/69, 87.0%), *repColE* (48/69, 69.6%), *repK* (43/69, 62.3%) and *repI1* (28/69, 41.0%). Other replicons (*repP, repFIA*, *repY*, *repN*, *repL/M* and *repHI2*) were detected in 2.9% to 17.4% of the isolates (Figure 2). Among the 45 Ec-MRnoB, 4 major replicon types were detected: *repFIB* (42/45, 93.3%), *repFII* (28/45, 62.2%), *repFIA* (24/45, 53.3%) and *repColE* (23/45, 51.0%). Furthermore, positive results were detected in some of these isolates for other replicons, including *repI1, repY, repP, repB/O*, *repA/C, repR* and *repK* (Figure 2).

Ec-ESBL and Ec-MRnoB isolates differed significantly in the presence of 5 replicons: *repK (p*<0.001*)*, *repFII (p*=0.002*)* and *repColE (p*<0.001*)* were more frequent among Ec-ESBL isolates, while *repFIA* (*p*<0.001) and *repA/C (p*=0.030*)* were more frequent among Ec-MRnoB isolates.

Nineteen ESBL-producing transconjugants were obtained from the 20 Ec-ESBL isolates selected for the conjugations assays (see Additional file 1). All transconjugants contained one or more plasmids. The more frequently detected replicons in the 19 transconjugants were: *repK* (14/19, 73.7%), *repFII* (11/19, 57.9%), *repI1* (5/19, 26.3%) and *repP* (2/19, 10.5%). With the three selective media used, transconjugants were obtained from 13 out of the 20 Ec-MRnoB isolates selected for conjugation assays (see Additional file 2). In all, 25 transconjugants were analysed, including 12 selected with ampicillin, which contained replicons *repI1* (n=6), *repFIB* (n=5), *repFII* (n=4) and *repFIA* (n=3), 10 selected with gentamicin [containing plasmids with replicons *repFIB* (n=6), *repFIA* (n=4) and r*epFII* (n=4)] and three selected with sulfamethoxazole, [with plasmids containing *repFIB* (n=2), *repFIA* (n=2) and *repFII* (n=2) replicons].

### Detection of resistance genes in wild-type strains and transconjugants

**Figure 3:**
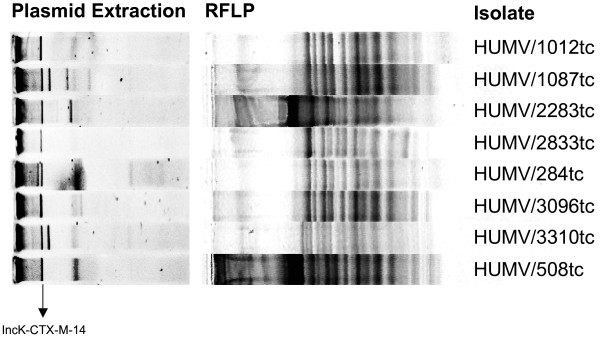
Restriction patterns to the IncK-CTX-M-14-plasmids belong to transconjugants obtained from the ESBL collection.

In the 69 Ec-ESBL isolates, genes coding for the following ESBL were detected: *bla*_*CTX-M-14*_ (51/69, 73.9%), *bla*_*SHV-12*_ (11/69, 15.9%), *bla*_*CTX-M-1*_ (6/69, 8.7%), *bla*_*SHV-2*_ (5/69, 7.2%), *bla*_*CTX-M-9*_ (2/69, 2.9%) and *bla*_*TEM-200*_ (1/69, 1.4%, first identified in this study). In addition, TEM-1 was detected in 33 (47.8%) isolates. No positive PCR results were obtained in this collection for genes coding for plasmid-mediated AmpC-β-lactamases. The ESBL genes produced by the 19 obtained transconjugants were: *bla*_*CTX-M-14*_ (16/19, 84.2%), *bla*_*CTX-M-9*_ (1/19, 5.3%), *bla*_*SHV-12*_ (1/19, 5.3%) and *bla*_*TEM-200*_ (1/19, 5.3%). Hybridization assays showed that the gene coding for CTX-M-14 was mobilizable by IncK plasmids. Moreover, the RFLP of plasmids from 13 IncK-CTX-M-14 showed the same restriction pattern in 8 isolates (Figure 3) (unfortunately, for the remaining 5 isolates the assay did not allow the production of a restriction pattern).

Amoxicillin resistance in the 45 Ec-MRnoB isolates was related to genes coding for TEM-1 (36/45, 80%), SHV-11 (2/45, 4.4%), SHV-1 (1/45, 2.2%) or OXA-1 (1/45, 2.2%). The relationship between amoxicillin resistance and TEM-1 production in these organisms was confirmed by detecting the corresponding gene in 24 out the 25 (96%) obtained transconjugants. The Ec-MRnoB resistant to both extended-spectrum cephalosporins and cefoxitin contained the gene coding for CMY-2, which was included in IncA/C plasmids, as confirmed by hybridization assays.

None of the 69 Ec-ESBL or the 45 Ec-MRnoB contained any of the studied plasmid-mediated quinolone resistance genes.

In the 69 Ec-ESBL isolates, class 1, class 2 or class 1 plus class 2 integrons were detected in 33.3%, 10.1% and 2.9% isolates, respectively (Table 3). Similarly, for the 45 Ec-MRnoB, positive results for class 1, class 2, and class 1 plus class 2 integrons were obtained for 75.6%, 4.4%, and 6.7% of the isolates (Table 4). The gene cassette arrays found in class 1 integrons for both *E. coli* collections are shown in Tables 3 and 4.

**Table 3:** **Distribution of gene cassette arrays found in class 1 integrons among phylogenetic groups of *E. coli *belongs to Ec-ESBL collection**

		**Phylogenetic groups (number of isolates)**			
**Cassettes**	**Total**	**A (N=23)**	**B1 (N=26)**	**B2 (N=5)**	**D (N=15)**
*ant(3″)-Ia*	3	2 (8.7%)	0	0	1 (6.7%)
*dfrA1-ant(3″)-Ia*	7	4 (17.4%)	2 (7.7%)	0	1 (6.7%)
*bla*_*OXA-1*_*-ant(3″)-Ia*	1	1 (4.3%)	0	0	0
*dfrA12-ant(3″)-Ib*	0	0	0	0	0
*dfrA16-ant(3″)-Ib*	4	2 (8.7%)	1 (3.8%)	0	1 (6.7%)
*dfrA17-ant(3″)-Ie*	8	3 (13%)	1 (3.8%)	1 (20%)	3 (20%)
*ant(2″)-Ia*	0	0	0	0	0
*ant(3″)-Ia-ant(2″)-Ia*	0	0	0	0	0

**Table 4:** **Distribution of gene cassette arrays found in class 1 integrons among phylogenetic groups of *E. coli *belongs to Ec-MRnoB collection**

		**Phylogenetic groups (number of isolates)**			
**Cassettes**	**Total**	**A (N=14)**	**B1 (N=9)**	**B2 (N=7)**	**D (N=15)**
*ant(3″)-Ia*	5	0	3 (33.3%)	1 (14.3%)	1 (6.7%)
*dfrA1-ant(3″)-Ia*	6	3 (21.4%)	1 (11.1%)	0	2 (13.3%)
*bla*_*OXA-1*_***-****ant(3″)-Ia*	1	1 (7.1%)	0	0	0
*dfrA12****-****ant(3″)-Ib*	3	1 (7.1%)	0	1 (14.3%)	1 (6.7%)
*dfrA16****-****ant(3″)-Ib*	0	0	0	0	0
*dfrA17****-****ant(3″)-Ie*	16	4 (28.6%)	2 (22.2%)	4 (57.1%)	6 (40%)
*ant(2″)-Ia*	1	0	0	0	1 (6.7%)
*ant(3″)-Ia****-****ant(2″)-Ia*	2	2 (14.3%)	0	0	0

## Discussion

This study presents comparative information about the microbiological characteristics of two groups of multiresistant clinical isolates of *E. coli* (producing or not producing ESBL, respectively), recovered in the same geographical and temporal context.

Analysis of Rep-PCR shows a wide clonal distribution among Ec-ESBL isolates and to a lesser extent among Ec-MRnoB isolates. This variability indicates that, in our area, multiresistance in *E. coli* is not always caused by the expansion of only one or a few clones, but it is often caused by the presence of multiple independent strains. The diversity of *E. coli* strains producing extended-spectrum beta-lactamase has been previously reported in a nationwide study in Spain [18]. In addition, MLST also showed evidences of small clusters of strains belonging to clonal complexes 354, 10 and 23 or to the sequence types 131, 224, 648 and 117. All these clonal groups have been previously described [19-21] as involved in the spread of certain genes coding for ESBLs and other resistance mechanisms.

Isolates belonging to the ST354Cplx have been related worldwide to the spread of ESBLs of the CTX-M family, associated with the presence of plasmids of different incompatibility groups [19,22]. In Spain, Mora et al. [19] have reported an increased prevalence of strains of ST354 producing CTX-M-14. However, in our study, the ST354 isolates do not produce an ESBL.

The ESBL-producing isolates of the ST10Cplx contained either IncK or IncI1 plasmids, as also described by other authors [23]. IncI1 plasmids have previously been identified in strains of human origin (both in patients and carriers) and in the commensal bacterial flora of diseased animals [24]. ST10Cplx isolates were also identified among non-ESBL detected in our study, but they did not contain IncI1 plasmids.

It has been previously demonstrated that *E. coli* O25:H4-ST131 is associated to the pandemic dissemination of the CTX-M-15 enzyme but this clone was also prevalent in healthy subjects from different European countries [1]. In a recent study on 100 consecutive extraintestinal *E. coli* isolates cultured in 2009, the ST131 clone represented 9% of all *E. coli* and about 25% of all multiresistant isolates in our centre [25]. In the current study, ST131 strains were also identified in both Ec-ESBL and Ec-MRnoB isolates.

CTX-M-14 was the most frequent ESBL identified in our Ec-ESBL isolates. In most cases the gene coding for this enzyme was in IncK plasmids and less frequently in an IncI1 plasmid, in agreement with a previous Spanish report [23]. Moreover, the IncK plasmids identified in this study showed identical restriction patterns (Figure 3), which suggest that the transmission of CTX-M-14 in our sanitary area is due to a specific plasmid belonging to this incompatibility group. Other reports have also shown the importance of CTX-M-14 in other countries, that was disseminated associated to IncFII plasmids [26,27].

The detection of both IncK and IncI1 plasmids in the Ec-MRnoB collection indicates that these mobile elements are not only important for ESBL dispersion, but may also be relevant for the transmission of other resistance mechanism, as suggested in previous reports [7]. On the other hand, resistance to expanded-spectrum cephalosporins associated to the production of the cephamycinase CMY-2 in the Ec-MRnoB was related to a different group of plasmids, namely those of the IncA/C group. IncA/C plasmids coding for CMY-2 have also been previously described in *E. coli* and *Salmonella enterica* isolates [7]. Moreover, 4 isolates were resistant to ceftazidime but they did not present plasmid-mediated AmpC β-lactamases, we presume that hyperproduction of AmpC was due to mutation in the promotor or the attenuator of the corresponding gene, as observed previously by others authors [28].

Plasmid typing showed that the dichotomous distribution of CTX-M-14 and CMY-2 among the two *E. coli* groups corresponded to an unequal distribution of two plasmid types associated to these enzymes: the A/C plasmids carrying CMY-2 were unique to the EcMRnoB group, while the IncK plasmids carrying CTX-M-14 were related to the Ec-ESBL group.

Interestingly, other plasmid species were common and highly represented in the two groups of isolates: IncF, ColE and IncI1. The high prevalence of IncF plasmids in both Ec-ESBL and Ec-MRnoB clearly indicates that this plasmid species is very well adapted in resistant *E. coli* strains independently of their resistance phenotype. A recent report has demonstrated that F replicons (FIA, FIB, FIC and FII) were the most frequently detected replicon types in *E. coli* strains producing or not producing ESBL [29]. Replicons of the IncF type were detected in 50% of *E. coli* from faeces of healthy, antibiotic-free humans and faecal flora from healthy birds in the USA, confirming that this plasmid type can be highly represented in *E. coli* populations also including susceptible strains [24]. Similarly, IncI1 plasmids have also been detected in *E. coli* from faecal flora of healthy humans and animals [24]. Finally, ColE plasmids are small, high copy number, not self-conjugative, producing bacteriocins, whose prevalence is not well estimated in recent collections of Enterobacteriaceae.

Several studies [30,31] have indicated that extraintestinal *E. coli* isolates are more commonly of phylogenetic groups B2 and D than of groups A and B1. In our series, groups D and B2 were more frequent in the Ec-MRnoB collection than in the Ec-ESBL. The decreased level of resistance among isolates of group B2 reported in some studies [32] was not observed in our case, as per definition all isolates were multiresistant. Although multiple studies indicate that use of fluoroquinolones is an independent factor for infections by multiresistant *E. coli*, plasmid-mediated quinolone resistance genes were not found among the isolates we have studied. The most likely explanation for quinolone resistance in our isolates is the presence of chromosomal mutations in their gyrase and topoisomerase IV genes.

In addition to the indicated resistance genes, we have found that the clinical isolates of multiresistant *E. coli* in our health area carry different classes of integrons. Ec-MRnoB showed a higher presence of these elements in comparison with the isolates belonging to the Ec-ESBL collection but, in both cases*,* the class 1 integrons containing *dfrA17-ant(3′)Ie* or *dfrA1-ant(3″)-Ia* genes were the most frequent ones. The implication of these elements in the spread of resistance in Spain [33] has been previously documented.

## Conclusion

In conclusion, this study has shown that, in our area, multiresistant *E. coli* producing either ESBL or other mechanisms of resistance are clonally diverse, although small clusters of related strains are also found. While both Ec-ESBL and EcMRnoB frequently contained IncFI plasmids, plasmids usually related to the most frequently detected ESBL (CTX-M-14), are uncommonly found in strains lacking this enzyme.

## Methods

### Bacterial isolates, susceptibility testing and clonal relationship

Two hundred multiresistant *E. coli* (one per patient) producing (n=100) or not producing ESBL (n=100), consecutively obtained between January 2004 and February 2005 at the Clinical Microbiology Service of the University Hospital Marqués de Valdecilla (Santander, Spain) were initially considered for this study. The organisms were obtained from urine (n=158) or from other samples (n=42, including 17 wound exudates, 8 samples from blood, 6 sputum, 6 naso-pharyngeal lavage, 2 catheter, 2 ascitic liquid and 1 bronchoalveolar aspirate). One hundred and sixteen isolates were from samples of patients admitted to the hospital and 84 from outpatients (database from Hospital Universitario Marqués de Valdecilla). No relevant differences were observed in the distribution of these parameters when comparing Ec-ESBL and Ec-MRnoB.

Identification and preliminary susceptibility testing (including ESBL production) of the isolates had been routinely performed with the WalkAway system (Dade Behring, Inc., West Sacramento, Ca., USA) using gram-negative MIC combo 1S panels. Confirmation of ESBL production and determination of MICs of imipenem, meropenem, aztreonam, piperacillin, cefoxitin, cefotetan, cefotaxime, cefotaxime-clavulanic acid, ceftazidime, ceftazidime-clavulanic acid and cefepime were performed using Dried MicroScan ESβL *plus* (Dade Behring, Inc., West Sacramento, Calif.) panels according to the manufacturer’s recommendations. In addition, susceptibility testing results of the following agents were confirmed by standardized microdilution, according to the CLSI standard [18]: amoxicillin (Sigma-Aldrich, Madrid, Spain), amoxicillin-clavulanic acid (GlaxoSmithKline, United Kingdom) (2:1), cefepime-clavulanic acid, piperacillin (Sigma-Aldrich)-tazobactam (Wyeth Pharmaceuticals, Madrid, Spain), amikacin (Sigma-Aldrich), gentamicin (Sigma-Aldrich), tobramycin (Sigma-Aldrich), nalidixic acid (Sigma-Aldrich), ciprofloxacin (Fluka, Madrid, Spain), tetracycline (Sigma-Aldrich), doxycycline (Sigma-Aldrich), and trimethoprim (Sigma-Aldrich)-sulfamethoxazole (Sigma-Aldrich) (19:1). Fixed concentrations of 4 mg/L of tazobactam or clavulanic acid were used in combination with piperacillin and cefepime, respectively. The results were interpreted according to the EUCAST breakpoints [17]. Isolates lacking ESBL were selected for this study if resistant to at least three of the following agents: amoxicillin, amoxicillin-clavulanic acid, nalidixic acid, gentamicin or tobramycin and trimethoprim-sulfamethoxazole. *E. coli* ATCC 25922 and *K. pneumoniae* ATCC 700603 were used as control strains in susceptibility testing assays. Phylogenetic grouping of the 200 isolates was determined by multiplex PCR, as described by Clermont et al. [19].

Clonal relationship was determined by Rep-PCR as previously described [20]. Amplicons were run in a 1.5% agarose gel for 100 min, stained with ethidium bromide (Sigma Chemical CO. St. Louis, USA) and photographed. Two isolates were considered to be clonally unrelated when at least two different bands were observed. Clonal relationship among isolates was also determined by *Xba*I-PFGE [21] when ESBL-producing isolates showed the same Rep-PCR pattern than isolates lacking ESBL, these isolates were also analysed by MLST, and this assay was also performed for 40 isolates selected for the conjugation assay representing the most frequent Rep-PCR patterns of each *E. coli* collection (see below). Detection by PCR and sequencing of 7 housekeeping genes (*gyrB, adk, purA, recA, icd, mdh* and *fumC*) were performed according to the *E. coli* MLST database (http://mlst.ucc.ie/mlst/dbs/Ecoli).

### Plasmid profile and hybridization experiments

After observing that some isolates with the same Rep-PCR pattern presented different clinical categories of at least two antimicrobial agents of different classes, 69 Ec-ESBL isolates and 45 Ec-MRnoB isolates were selected for plasmid analysis and detection of plasmid-mediated genes coding for resistance to β-lactams and quinolones, according to the following criteria: a) all isolates from each Rep-PCR pattern with single isolates, b) one isolate of each antimicrobial susceptibility pattern from Rep-PCR patterns containing >=2 isolates.

Plasmid DNA was extracted by the Kado-Liu method [22] and separated on 0.9% horizontal agarose gels electrophoresis. Plasmids R27 (169 kb, Genbank access AF250878), R1 (94 kb, Genbank access NC_003277), RP4 (55 kb, [23]), and ColE1 (6 kb, Genbank access J01566) were used as size standards.

Plasmids were also characterized by PCR-based replicon typing (PBRT), as described elsewhere, using the respective PBRT controls [4,5]. The obtained amplicons were sequenced to confirm their identity.

Plasmids were transferred onto nylon membranes by southern blotting (Roche, Mannheim, Germany). Amplicons from *bla*_*CTX-M-14*_ and *bla*_*CMY-2*_ were obtained by PCR using DNA from *E. coli* CCG02 and *E. coli* B-12 [24], respectively. Similarly, plasmids R387 and pIP40a [5] were used to obtain PCR amplicons from *repK* and *repA/C*, respectively. DNA probes prepared with DIG-High Prime (Roche, Penzberg, Germany) were used to investigate the presence of *bla*_*CTX-M-14*_ and *repK* genes in the same plasmid of Ec-ESBL isolates and of *bla*_*CMY-2*_ and *repA/C* genes in the same plasmid of Ec-MRnoB isolates.

In 13 transconjugants of the belonging to ESBL collection the relationship among *repK*-*CTX-M-14*-plasmids was determined by comparison of their DNA patterns generated after digestion with the *Eco*RI and *Pst*I enzymes and electrophoresis in 1.5% agarose, as described elsewhere [25].

### Conjugation assays

**Figure 4:**
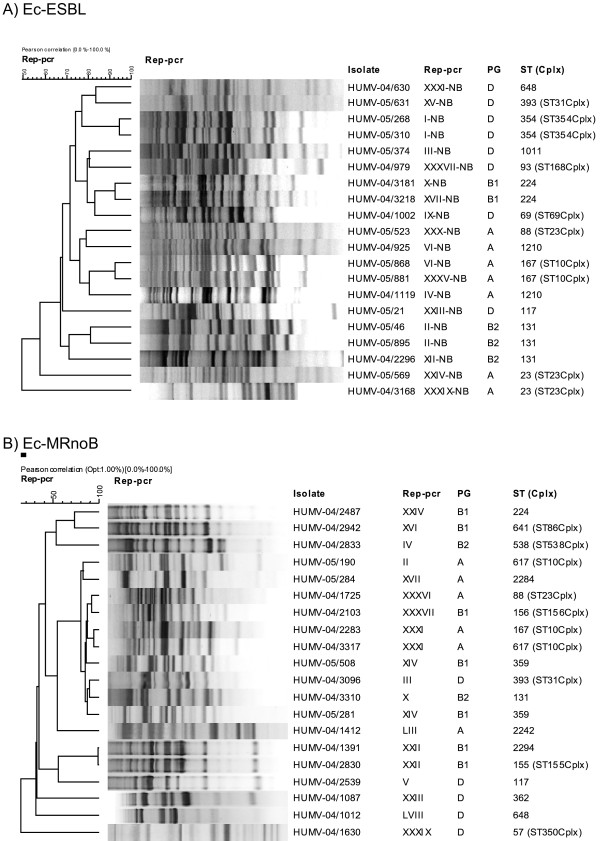
**Clonal relationship between isolates selected for conjugation assays in both *E. coli *collections. A**) Ec-ESBL, **B**) Ec-MrnoB.

Conjugation assays were performed with 20 Ec-ESBL and 20 Ec-MRnoB, which are representative of the most common Rep-PCR/antibiotic resistance patterns (Figure 4). *E. coli* J53 resistant to sodium azide was used as a recipient strain. Transconjugants from the Ec-ESBL isolates were selected with sodium azide (100 mg/L) plus cefotaxime (2 mg/L), while for the Ec-MRnoB, transconjugants were selected on three different media: sodium azide (100 mg/L) plus ampicillin (100 mg/L), gentamicin (8 mg/L) or sulfamethoxazole (1000 mg/L).

### Detection of resistance determinants

Five multiplex PCRs (Table 5) were performed using previously published conditions to detect genes that are usually included in conjugative plasmids: *bla*_*TEM*_*, bla*_*SHV*_*, bla*_*OXA-1*_ and *bla*_*PSE-1*_[26], plasmid-mediated AmpC-type enzymes [27], *bla*_*CTX-M*_ β-lactamases [26], plasmid-mediated quinolone-resistance genes, including *qnrA*, *qnrB*, *qnrS*, *aac(6′)-Ib-cr* and *qepA*[28] and tetracyclines-resistance genes *tet*(A), *tet*(B) and *tet*(G) [26]. The identity of the complete genes detected by the multiplex PCR was confirmed by specific PCR (using appropriate primers) and sequencing of the two DNA strands. Finally, class 1 and class 2 integrons were detected by PCR (Table 5) and the variable regions of class 1 integrons were sequenced using specific primers for the 3′CS and 5′CS ends as described elsewhere [29].

**Table 5:** PCR conditions used for detection of antimicrobial resistance genes

**Resistance**	**Gene**	**Primers**	**Tm (°C)**	**Amplicon size (pb)**
β-Lactams	*bla*_*TEM-1*_	5′-ttgggtgcacgagtgggt-3′	60	503
		5′-taattgttgccgggaagc-3′		
	*bla*_*oxa-1*_	5′-agcagcgccagtgcatca-3′		708
		5′-attcgaccccaagtttcc-3′		
	*bla*_*pse-1*_	5′-cgcttcccgttaacaagtac-3′		419
		5′-ctggttcatttcagatagcg-3′		
	*bla*_*shv*_	5′-tcgggccgcgtaggcatg-3′		606
		5′-agcagggcgacaatcccg-3′		
Quinolones	*qnrA*	5′-agaggatttctcacgccagg-3′	56	579
		5′-tgccaggcacagatcttgac-3′		
	*qnrB*	5′-ggmathgaaattcgccactg-3′		263
		5′-ttygcbgyycgccagtcggcg-3′		
	*qnrS*	5′-gcaagttcattgaacagggt-3′		427
		5′-tctaaaccgtcgagttcggcg-3′		
	*qepA*	5′-aactgcttgagcccgtagat-3′		189
		5′-cgtgttgctggagttcttcc-3′		
	*aac(6′)-Ib-cr*	5′-ttgcaatgctgaatggagag-3′		218
		5′-cgtttggatcttggtgacct-3′		
Cephalosporins	*bla*_*ctx-m-like*_	5′-cgatgtgcagtaccagtaa-3′	60	585
		5′-ttagtgaccagaatcagcgg-3′		
	*bla*_*ctx-m-group1*_	5′-atggttaaaaaatcactgcg-3′		876
		5′-ttacaaaccgtcggtgac-3′		
	*bla*_*ctx-m-group9*_	5′-atggtgacaaagagagtgcaac-3′		876
		5′-ttacagcccttcggcgatg-3′		
	*bla*_*ctx-m-group2*_	5′-tcaagaagagcgacctggtt-3′		601
		5′-gatacctcgctccatttattg-3′		
Cephamycinases	*bla*_*acc*_	5′-aacagcctcagcagccggtta-3′	64	346
		5′-ttcgccgcaatcatccctagc-3′		
	*bla*_*cit*_	5′-tggccagaactgacaggcaaa-3′		462
		5′-tttctcctgaacgtggctggc-3′		
	*bla*_*dha*_	5′-aactttcacaggtgtgctgggt-3′		405
		5′-ccgtacgcatactggctttgc-3′		
	*bla*_*mox*_	5′-gctgctcaaggagcacaggat-3′		520
		5′-cacattgacataggtgtggtg-3′		
	*bla*_*ebc*_	5′-tcggtaaagccgatgttgcgg-3′		302
		5′-cttccactgcggctgccagtt-3′		
	*bla*_*fox*_	5′-aacatggggtatcagggagatg-3′		190
		5′-caaagcgcgtaaccggattgg-3′		
Tetracyclines	*tet (G)*	5′-gctcggtggtatctctgc-3′	60	500
		5′-agcaacagaatcgggaac-3′		
	*tet (A)*	5′-gctacatcctgcttgcct-3′		210
		5′-catagatcgccgtgaaga-3′		
	*tet (B)*	5′-ttggttaggggcaagttttg-3′		600
		5′-gtaatgggccaataacaccg-3′		
Integrons	*intI1*	5′-gccttgctgttcttctac-3′	55	558
		5′-gatgcctgcttgttctac-3′		
	*intI2*	5′-cacggatatgcgacaaaaaggt-3′	65	788
		5′-gtagcaaacgagtgacgaaatg-3′		
	Int-class 1 (IC1)	5′-ggcatccaagcagcaagc-3′	58	variable
		5′-aagcagacttgacctgat-3′		
	Int-class 2 (IC2)	5′-cgggatcccggacggcatgcacgatttgta-3′	65	variable
		5′-gatgccatcgcaagtacgag-3′		

### Statistical methods

The proportions of Ec-ESBL and of Ec-MRnoB isolates containing different replicon elements were compared by the chi-square test. Differences were considered significant for p<0.05. The test was performed using the SPSS 11.0 program.

## Competing interest

Luis Martínez.-Martínez: reports that he has been a consultant for Wyeth and Pfizer, has served as speaker for Wyeth, Merck, Pfizer, and Janssen-Cilag, and has received research support from Merck, Wyeth, and Janssen-Cilag. The other authors declare that they have no competing interests.

## Authors’ contributions

BRdC carried out the molecular genetic studies, participated in the sequence alignment and drafted the manuscript. EJR carried out the molecular genetic studies. LV and CT participated in the design of the study and performed the statistical analysis. BG, AC and LMM conceived the study, and participated in its design and coordination and helped to draft the manuscript. All authors read and approved the final manuscript.

## Supplementary Material

Additional file 1 Resistance phenotype and resistance genes in 19 EcESBL and their derived transconjugants. Click here for file

Additional file 2 Resistance phenotype and resistance genes in 13 Ec-MRnoB and their derived transconjugants. Click here for file
