# Supplementary material for: Molecular characterization of multiresistant Escherichia coli producing or not extended-spectrum β-lactamases
Source: BMC Microbiol. 2013 Apr 16;13:84. doi: 10.1186/1471-2180-13-84 (PMC3637601; doi:10.1186/1471-2180-13-84)
Supplement: Additional file 1 — Resistance phenotype and resistance genes in 19 EcESBL and their derived transconjugants. [file 1471-2180-13-84-S1.doc]

**Additional file 1:** Resistance phenotype and resistance genes in 19 EcESBL and their derived transconjugants.

| **Isolate** | **Origin^a^** | **ST (Cplx)*** | Resistance phenotype^ | **Resistance genes** | Replicon-typing |
| --- | --- | --- | --- | --- | --- |
| HUMV-05/190 | Outpatient  (Centre 1) | 617 (ST10Cplx) | ESBL, NAL, CIP, TET, SXT | *bla_CTX-M-14_, tet(A), dfrA1, sul1, sul2* | FIB, FII, K |
| HUMV/190tc |  |  | ESBL | *bla_CTX-M-14_* | K |
| HUMV-05/281 | Outpatient  (Centre 2) | 359 | ESBL, NAL, CIP, TET | *bla_CTX-M-14_, tet(B), sul2* | FIB, FII, K, ColE |
| HUMV/281tc |  |  | ESBL | *bla_CTX-M-14_* | K, FII |
| HUMV-05/284 | Inpatient  (HUMV) | 2295 | ESBL, GEN, TOB, NAL, CIP, TET | *bla_CTX-M-14_, tet(B), aac(3)-II* | FIB, FIA, FII, K |
| HUMV/284tc |  |  | ESBL | *bla_CTX-M-14_* | K |
| HUMV-05/508 | Outpatient  (Centre 3) | 359 | ESBL, NAL, CIP, TET | *bla_CTX-M-14_, tet(A)* | FIB, FII, K, ColE |
| HUMV/508tc |  |  | ESBL | *bla_CTX-M-14_* | K, FII |
| HUMV-04/1012 | Outpatient  (Centre 4) | 648 | ESBL, NAL, CIP, TET | *bla_CTX-M-14_, bla_TEM-1_, tet(A), tet(B), sul2* | K |
| HUMV/1012tc |  |  | ESBL | *bla_CTX-M-14_* | K |
| HUMV-04/1087 | Inpatient  (HUMV) | 362 | ESBL, NAL, CIP, TET, SXT | *bla_CTX-M-14_, bla_TEM-1_, tet(A), dfrA1, sul1, sul2* | FIB, FII, K, I1-I, ColE |
| HUMV/1087tc |  |  | ESBL | *bla_CTX-M-14_* | K |
| HUMV-04/1412 | Inpatient  (HUMV) | 2292 | ESBL, NAL, CIP, TET | *bla_SHV-12_, tet(A), sul1* | FIB, FII, P, I1-I, ColE |
| HUMV/1412tc |  |  | ESBL, TET | *bla_SHV-12_, tet(A)* | I1-I, P |
| HUMV-04/1630 | Inpatient  (HUMV) | 57 (ST350Cplx) | ESBL, GEN, TOB, NAL, CIP, TET | *bla_TEM-200_, tet(B)* | FIB, FII, B/O |
| HUMV/1630tc |  |  | ESBL, GEN, TOB | *bla_TEM-200_* | FIB |
| HUMV-04/1725 | Outpatient  (Centre 5) | 88 (ST23Cplx) | ESBL, TET, SXT | *bla_CTX-M-14_, bla_TEM-1_, tet(A), dfrA1, sul1* | FIB, FII, K, ColE |
| HUMV/1725tc |  |  | ESBL | *bla_CTX-M-14_* | K, FIB, FII, ColE |
| HUMV-04/2103 | Outpatient  (Centre 6) | 156 (ST156Cplx) | ESBL, NAL, CIP, SXT | *bla_CTX-M-14_, tet(A), dfrA14* | FIB, FII, K, ColE |
| HUMV/2103tc |  |  | ESBL | *bla_CTX-M-14_* | K |
| HUMV-04/2283 | Inpatient  (HUMV) | 167 (ST10Cplx) | ESBL, GEN, TOB, NAL, CIP, TET, SXT | *bla_CTX-M-14_, bla_TEM-1_, tet(B), dfrA17, aac(3)-II, sul1, sul2* | FIA, FIB, FII, K, ColE |
| HUMV/2283tc |  |  | ESBL | *bla_CTX-M-14_* | K |
| HUMV-04/2487 | Outpatient  (Centre 7) | 224 | ESBL, NAL, CIP, TET | *bla_CTX-M-14_, bla_TEM-1_, tet(A)* | FIB, FII, I1-I |
| HUMV/2487tc |  |  | ESBL | *bla_CTX-M-14_* | I1-I |
| HUMV-04/2539 | Outpatient  (Centre 8) | 117 | ESBL, NAL, TET, SXT | *bla_CTX-M-9_, tet(A), dfrA16, sul1, sul2* | FIB, FII, P, I1-I, ColE |
| HUMV/2539tc |  |  | ESBL, TET, SXT | *bla_CTX-M-9_, tet(A), dfrA16, sul1* | I1-I, P, ColE |
| HUMV-04/2830 | Outpatient  (Centre 9) | 155 (ST155Cplx) | ESBL, NAL, CIP, SXT | *bla_CTX-M-14_, bla_TEM-1_, tet(B), dfrA1* | FIB, FII, K, I1-I, ColE |
| HUMV/2830tc |  |  | ESBL | *bla_CTX-M-14_* | K |
| HUMV-04/2833 | Outpatient  (Centre 4) | 538 (ST538Cplx) | ESBL, NAL, CIP, TET, SXT | *bla_CTX-M-14_, tet(A), dfrA1* | FIB, FIA, FII, K, ColE |
| HUMV/2833tc |  |  | ESBL | *bla_CTX-M-14_* | K, ColE |
| HUMV-04/2942 | Inpatient  (HUMV) | 641 (ST86Cplx) | ESBL, NAL, TET | *bla_CTX-M-14_, bla_TEM-1_, tet(A)* | FIB, FII, K, ColE |
| HUMV/2942tc |  |  | ESBL, TET | *bla_CTX-M-14_, bla_TEM-1_, tet(A)* | FIB, K, FII |
| HUMV-04/3096 | Inpatient  (HUMV) | 393 (ST31 Cplx) | ESBL, NAL, CIP, TET, SXT | *bla_CTX-M-14_, bla_TEM-1_, tet(A), tet(B), dfrA17, sul1, sul2* | FIA, K, ColE |
| HUMV/3096tc |  |  | ESBL | *bla_CTX-M-14_* | K |
| HUMV-04/3310 | Inpatient  (HUMV) | 131 | ESBL, NAL, CIP | *bla_CTX-M-14_, tet(A), sul1* | FIA, FII, K, I1-I |
| HUMV/3310tc |  |  | ESBL | *bla_CTX-M-14_* | I1-I, K, FII |
| HUMV-04/3317 | Outpatient  (Centre 10) | 617 (ST10Cplx) | ESBL, NAL, CIP, TET | *bla_CTX-M-14_, bla_TEM-1_, tet(A), tet(B)* | FIB, FII, I1-I, ColE |
| HUMV/3317tc |  |  | ESBL | *bla_CTX-M-14_, bla_TEM-1_* | FIB, I1-I, ColE |

a: HUMV= Hospital Universitario Marqués de Valdecilla. *Cplx: clonal complex. ^ESBL= extended-spectrum β-lactamases; GEN= gentamicin, TOB=tobramycin, NAL=nalidixic acid, CIP=ciprofloxacin, TET= tetracycline, SXT= trimethoprim-sulfamethoxazole.
