# Supplementary material for: Molecular characterization of multiresistant Escherichia coli producing or not extended-spectrum β-lactamases
Source: BMC Microbiol. 2013 Apr 16;13:84. doi: 10.1186/1471-2180-13-84 (PMC3637601; doi:10.1186/1471-2180-13-84)
Supplement: Additional file 2 — Resistance phenotype and resistance genes in 13 Ec-MRnoB and their derived transconjugants. [file 1471-2180-13-84-S2.doc]

**Additional file 2:** Resistance phenotype and resistance genes in 13 Ec-MRnoB and their derived transconjugants.

| **Isolate** | **Origin^a^** | **ST (Cplx*)** | Resistance phenotype^ | Resistance genes | **Replicon-typing** |
| --- | --- | --- | --- | --- | --- |
| HUMV-05/21 | Inpatient  (HUMV) | 117 | AMX, GEN, TOB, NAL, TET, SXT | *bla_TEM-1_, tet(B), dfrA1, sul2* | FIB, FII, I1-I, ColE |
| HUMV21tcA |  |  | AMX, GEN | *bla_TEM-1_* | I1-I, ColE |
| HUMV21tcG |  |  | AMX, GEN | *bla_TEM-1_* | I1-I, ColE |
| HUMV-05/46 | Outpatient  (Centre 11) | 131 | AMX, GEN, TOB, NAL, CIP, TET, SXT | *bla_TEM-1_, tet(A), sul1, sul2* | FIB, FIA, FII, I1-I, ColE |
| HUMV46tcA |  |  | AMX, GEN, TOB, TET, SXT | *bla_TEM-1_, tet(A), sul1, sul2* | FIB, I1-I, ColE |
| HUMV46tcG |  |  | AMX, GEN, TOB, TET, SXT | *bla_TEM-1_, tet(A), sul1, sul2* | FIB, FII, ColE |
| HUMV46tcS |  |  | AMX, GEN, TOB, TET, SXT | *bla_TEM-1_, tet(A), sul1, sul2* | FIB, I1-I, ColE |
| HUMV-05/268 | Inpatient  (HUMV) | 354 (ST354Cplx) | AMX, GEN, TOB, NAL, CIP, TET, SXT | *bla_TEM-1_, tet(A), tet(B), dfrA1, dfrA17, sul1, sul2* | FIB, FIA, P, K, ColE |
| HUMV268tcG |  |  | TET | *tet(A)* | FIB, P, K, ColE |
| HUMV-05/310 | Outpatient  (Centre 7) | 354 (ST354Cplx) | AMX, GEN, TOB, NAL, CIP, SXT | *bla_TEM-1_, dfrA17, sul1* | FIB, FIA, FII, I1-I, ColE |
| HUMV310tcA |  |  | AMX | *bla_TEM-1_* | I1-I, ColE |
| HUMV-05/895 | Inpatient  (HUMV) | 131 | AMX, FOX, GEN, TOB, NAL, CIP, TET, SXT | *bla_CMY-2_,, bla_TEM-1_, tet(A), sul1, sul2* | FIB, FIA, FII, A/C, ColE |
| HUMV895tcA |  |  | AMX, GEN, TET, SXT | *bla_TEM-1_, tet(A), sul1, sul2* | FIB, FIA, FII |
| HUMV895tcG |  |  | AMX, GEN, TET, SXT | *bla_TEM-1_, tet(A), sul1, sul2* | FIB, FIA, FII |
| HUMV895tcS |  |  | AMX, GEN, TET, SXT | *bla_TEM-1_, tet(A), sul1, sul2* | FIB, FIA, FII, ColE |
| HUMV-05/868 | Inpatient  (HUMV) | 167 (ST10Cplx) | AMX, GEN, TOB, NAL, CIP, TET, SXT | *bla_CMY-2_, bla_TEM-1_, tet(A), tet(B), sul1, sul2* | FIA, FIB, FII, ColE |
| HUMV868tcA |  |  | AMX, GEN, TET | *bla_TEM-1_, tet(B), sul 1* | FIA, FIB, FII |
| HUMV868tcG |  |  | AMX, GEN, TET | *bla_TEM-1_, tet(B), sul 1* | FIA, FIB, FII |
| HUMV-05/881 | Outpatient  (Centre 10) | 167 (ST10Cplx) | AMX, GEN, TOB, NAL, TET, SXT | *bla_TEM-1_, tet(B), sul1, sul2* | FIA, FIB, FII, ColE |
| HUMV/881tcA |  |  | AMX, GEN, TET, SXT | *bla_TEM-1_, tet(B), sul1* | FIA, FIB, FII, ColE |
| HUMV/881tcG |  |  | AMX, GEN, TET | *bla_TEM-1_, tet(B)* | FIA, FIB, FII, ColE |
| HUMV-04/925 | Inpatient  (HUMV) | 1210 | AMX, GEN, TOB, NAL, CIP, TET, SXT | *bla_OXA-1_, bla_TEM-1_, tet(B), dfrA1, dfrA4/14, aac(3)-II* | FIA, FIB, FII, B/O, ColE |
| HUMV925tcA |  |  | AMX, TET | *bla_TEM-1_, tet(B)* | B/O |
| HUMV925tcS |  |  | AMX, TET | *bla_TEM-1_, tet(B)* | FIA/FIB, FII |
| HUMV-04/979 | Inpatient  (HUMV) | 93 (ST168Cplx) | AMX, GEN, TOB, NAL, TET, SXT | *bla_TEM-1_, tet(A), dfrA1, sul1* | FIB, FII, I1-I, B/O, ColE |
| HUMV979tcA |  |  | AMX, GEN, TOB | *bla_TEM-1_* | I1-I |
| HUMV979tcG |  |  | AMX, GEN, TOB | *bla_TEM-1_* | I1-I |
| HUMV-04/1119 | Inpatient  (HUMV) | 1210 | AMX, TOB, NAL, CIP, SXT | *bla_TEM-1_, dfrA1, sul2, aac(3´)-IV* | I1-I, ColE |
| HUMV1119tcA |  |  | AMX | *bla_TEM-1_* | I1-I |
| HUMV-04/2296 | Inpatient  (HUMV) | 131 | AMX, CTX, CAZ, FOX, GEN, TOB, NAL, TET | *bla_CMY-2_, bla_TEM-1_, tet(A)* | FIB, FII, I1-I, A/C, ColE |
| HUMV-04/2296tcA |  |  | AMX | *bla_TEM-1_* | FIB, FII, ColE |
| HUMV-04/2296tcG |  |  | AMX, CTX, CAZ, FOX, TET | *bla_CMY-2_,, bla_TEM-1_, tet(A), sul2* | A/C, ColE |
| HUMV-04/3181 | Inpatient  (HUMV) | 224 | AMX, FOX, GEN, TOB, NAL, CIP, TET, SXT | *bla_CMY-2_, bla_TEM-1_, tet(A)* | FIB, FII, A/C, ColE |
| HUMV-04/3181tcA |  |  | AMX, FOX, GEN, TOB, TET, SXT | *bla_CMY-2_, bla_TEM-1_, tet(A)* | A/C |
| HUMV-04/3181tcG |  |  | AMX, FOX, GEN, TOB, TET, SXT | *bla_CMY-2_, bla_TEM-1_, tet(A)* | A/C |
| HUMV-04/3218 | Inpatient  (HUMV) | 224 | AMX, FOX, GEN, TOB, NAL, CIP, TET, SXT | *bla_CMY-2_, bla_TEM-1_, tet(A), strA/dfrA14/strB, sul2* | FIA, FIB, FII, I1-I, ColE |
| HUMV3218tcA |  |  | AMX, FOX | *bla_CMY-2_, bla_TEM-1_* | I1-I |
| HUMV3218tcG |  |  | AMX | *no detected* | I1-I, FIB, FIA |

a: HUMV= Hospital Universitario Marqués de Valdecilla. *Cplx: clonal complex. ^AMX=amoxicillin; CAZ= ceftazidime, FOX= cefoxitin; CTX: cefotaxime; GM= gentamicin, TO=tobramycin, NAL=nalidixic acid, CIP=ciprofloxacin, TET= tetracycline, SXT= trimethoprim-sulfamethoxazole; tcA: transconjugant obtained in ampicillin medium; tcG: transconjugant obtained in gentamicin medium. tcS: transconjugant obtained in sulfamethoxazole medium.
